# Overlap of copper and iron uptake systems in mitochondria in *Saccharomyces cerevisiae*

**DOI:** 10.1098/rsob.150223

**Published:** 2016-01-13

**Authors:** Katherine E. Vest, Jing Wang, Micah G. Gammon, Margaret K. Maynard, Olivia L. White, Jai A. Cobine, Wilkerson K. Mahone, Paul A. Cobine

**Affiliations:** 1 Department of Biological Sciences, Auburn University, Auburn, AL 36849, USA; 2 College of Charleston, Charleston, SC 29424, USA

**Keywords:** copper, iron, cytochrome *c* oxidase, mitochondrial carrier family

## Abstract

In *Saccharomyces cerevisiae*, the mitochondrial carrier family protein Pic2 imports copper into the matrix. Deletion of *PIC2* causes defects in mitochondrial copper uptake and copper-dependent growth phenotypes owing to decreased cytochrome *c* oxidase activity. However, copper import is not completely eliminated in this mutant, so alternative transport systems must exist. Deletion of *MRS3*, a component of the iron import machinery, also causes a copper-dependent growth defect on non-fermentable carbon. Deletion of both *PIC2* and *MRS3* led to a more severe respiratory growth defect than either individual mutant. In addition, *MRS3* expressed from a high copy number vector was able to suppress the oxygen consumption and copper uptake defects of a strain lacking *PIC2*. When expressed in *Lactococcus lactis*, Mrs3 mediated copper and iron import. Finally, a *PIC2* and *MRS3* double mutant prevented the copper-dependent activation of a heterologously expressed copper sensor in the mitochondrial intermembrane space. Taken together, these data support a role for the iron transporter Mrs3 in copper import into the mitochondrial matrix.

## 1. Introduction

Copper is an essential trace element used in various pathways, including iron acquisition, respiration and detoxification of reactive oxygen species. Because of its potential toxicity, the import, localization and storage of copper is tightly controlled. Cells use a combination of low-affinity and high-affinity transport systems to bring copper into the cytoplasm [1,2]. Inside the cell, protein and small molecule chaperones sequester copper and deliver it to target enzymes. The chaperone Atx1 carries copper to the P-type ATPase Ccc2 in the trans-Golgi network for incorporation into the multicopper oxidase Fet3, which is required for high affinity iron uptake [3]. Copper is delivered to the Cu, Zn superoxide dismutase (Sod1) by its chaperone Ccs1 in the cytosol and in the mitochondrial intermembrane space (IMS) [4,5]. Currently, no protein or small molecule has been unambiguously identified in recruitment of copper to mitochondria [6].

In addition to Sod1, mitochondrial copper is used by cytochrome *c* oxidase (CcO), the final enzyme complex in the electron transport chain [7]. The CcO complex localizes to the inner membrane (IM), and copper cofactor insertion must be tightly coordinated with its assembly; a series of chaperone proteins add copper as the individual subunits are translated and inserted into the IM [8]. The soluble IMS protein Cox17 delivers copper to the IM proteins Cox11 and Sco1, which assemble the Cu_B_ site in the Cox1 subunit and the Cu_A_ site in the Cox2 subunit, respectively [9–11]. Sco1 has an additional role in regulating cellular copper concentration and mutations in *SCO1* cause defects in both import and export of copper at the plasma membrane [12,13]. While CcO is the major mitochondrial copper enzyme, the bulk of mitochondrial copper is found in a labile pool [14–18]. The identity of the ligand (or ligands) for the labile copper pool remains unknown. Investigations with matrix-targeted copper-binding proteins and matrix-targeted fluorescent sensors suggest that the labile copper pool is present within the mitochondrial matrix [14,18]. Copper is transported into the mitochondrial matrix by the mitochondrial carrier family (MCF) protein Pic2 [19]. The phenotypes of a *pic2Δ* mutant and the phenotypes of yeast cells expressing heterologous copper-binding proteins in the matrix suggest that this matrix copper is redistributed to the IMS for assembly into CcO and mitochondrial Sod1 [19,20].

The MCF proteins are involved in translocation of TCA intermediates, nucleoside di- and triphosphates, and other substrates across the mitochondrial IM [21]. MCF proteins have a basic structure consisting of three pseudo-symmetric repeats of approximately 100 amino acids that contain two transmembrane (TM) helices connected by a loop with a short α-helix. The TM helices contain a conserved PX(D/E)XX(R/K) motif that is a signature of all MCF proteins [22]. These motifs form salt bridges important for the transition between the open and closed states. Using a single binding-gated pore mechanism, the interconversion between these states is required for transport [22]. Residues required for transport, therefore, have symmetry, with any apparent asymmetric residues being responsible for substrate binding and determining the requirement for counter substrates, co-substrates and the directionality of transport [22]. Identification of symmetric and asymmetric residues has allowed for computational prediction of substrates without knowledge of the protein structure [22].

Multiple MCF proteins are known to play a role in mitochondrial metal homeostasis. Importantly, we recently demonstrated that Pic2 acts as a copper importer in yeast [19]. Mrs3, Mrs4 and their metazoan homologues are responsible for high-affinity iron transport across the IM [23]. Deletion of both *MRS3* and *MRS4* caused a severe growth defect in yeast grown under iron-depleted conditions, whereas mutation of metazoan mitoferrin was embryonic lethal owing to severe anaemia [24,25]. In addition to Mrs3 and Mrs4 in yeast, Rim2 was shown to mediate transport of nucleotide-bound iron across the IM [26]. Still other MCF proteins, such as Mtm1 and Ggc1, have been shown to have an indirect effect on mitochondrial iron homeostasis [27–29]. The cases of Mtm1 and Ggc1 reveal that MCFs can indirectly modify metal-related phenotypes in yeast, highlighting the need for both phenotypic characterization and biochemical assessment.

Pic2 cannot be the only protein involved in mitochondrial copper import as deletion of the *PIC2* gene caused a respiratory growth defect only under copper-limiting conditions [19]. Mitochondria from *pic2Δ* mutants still had 40–70% of wild-type copper levels and could still import copper, though at a lower capacity than those from wild-type cells [19]. Pic2 is homologous to the mitochondrial phosphate carriers identified in other eukaryotes [30], but in *Saccharomyces cerevisiae* Mir1 is the major phosphate carrier. In a comprehensive computational analysis of the residues involved in substrate binding and transport, Pic2, Mir1 and the other phosphate carriers from humans are a phylogenetically unique group [22]. However, the authors note that they share similarity with Mrs3 in two regions predicted to mediate substrate binding [22], including one of the three histidine residues that were recently shown to be involved in iron transport [31]. Here, we have used phenotypic and biochemical assays to show that the iron carrier Mrs3 is also involved in the mitochondrial copper import pathway.

## 2. Material and methods

### 2.1. Yeast strains, culture conditions and standard methods

Yeast strains used were BY4741 (*MATa, leu2Δ, met15Δ, ura3Δ, his3Δ*) and isogenic kanMX4 mutant from Open Biosystems (Huntsville, AL). Double mutants were constructed by homologous recombination of the *URA3MX* cassette at the *MRS3* locus. Cultures were grown in 1% yeast extract, 2% peptone (YP) medium or in synthetic defined media with selective amino acids excluded and with the appropriate carbon source added. Bio101 yeast nitrogen base plus 0.1 mM ferrous sulfate was used to give copper-deficient conditions. If required, extracellular copper was depleted with bathocuproine sulfonate (BCS) or silver was added as a mitochondrial copper competitor. Exogenous copper was supplied as CuSO_4_. Growth tests were performed at 30°C with 1 in 10 serial dilutions of overnight pre-cultures grown in YP plus 1% glucose.

IM-hSOD1 was constructed by inserting the hSod1 open reading frame (ORF) in-frame with the sequence encoding the N-terminal 104 residues of Sco2 (YBR024W) that encode a previously described mitochondrial targeting sequence and a TM domain [20]. This construct was then integrated in the yeast genome at the *CCS1* locus to create *ccs1Δ::IMhSOD1* (α, *can1Δ::STE2pr-Sp_his5 lyp1Δ* *his3Δ* *leu2Δ* *ura3Δ* *met15Δ*) [32]*.* Constructs were verified by dideoxynucleotide sequencing prior to use.

### 2.2. Fractionation of mitochondrial copper pool

Preparation and fractionation of mitochondria was performed as previously described [14]. To obtain the matrix copper pool, cells were extracted in 100% methanol. Resulting extracts were dried and resuspended in water. CuL was prepared using Whatman DE52 anion exchange resin, which was washed in 10 mM ammonium acetate (pH 8.0). Ligand fractions were eluted using two bed volumes of 1 M ammonium acetate, pH 8.0. These were dried and washed in water before being loaded onto a Sonoma C18 column and separated by a 0–100% methanol gradient for 30 min on a Shimadzu UFLC. Fractions were collected and analysed for fluorescence using an excitation maximum of 220 nm and emission maximum of 360 nm or an excitation maximum of 320 nm with an emission maximum of 400 nm (PerkinElmer Life Sciences LS55 spectrofluorimeter) and for copper by ICP-OES (PerkinElmer Life Sciences 9300-DV).

### 2.3. Expression of Mrs3 in *Lactococcus lactis*

*Lactococcus lactis* cells transformed with vector (pNZ8148) alone or pNZ8148 (MoBiTec) carrying the *PIC2* or *MRS3* gene were grown overnight at 30°C in M17 medium with 0.5% glucose and 10 µg ml^−1^ chloramphenicol. Cells were diluted into fresh medium at an OD_600_ of 0.1, grown to an OD_600_ of 0.4 and induced using 1 ng ml^−1^ nisin for 5 h or overnight. To determine silver toxicity in *L. lactis* strains containing vector, *PIC2* or *MRS3*, cells were grown in a 96-well plate containing M17 medium plus 1 ng ml^−1^ nisin and increasing concentrations of silver (0–250 µM). Iron chelation was achieved by addition of 100 µM bathophenanthroline disulfonate (BPS). Controls containing M17 without nisin or M17 plus silver without nisin were included. Optical density at 600 nm was used to assess growth after 18 h at 30°C.

### 2.4. Copper uptake assay

Isolated mitochondria suspended in 0.6 M sorbitol were incubated with CuL for 60-s intervals and removed from solution by centrifugation. Uptake was measured by ICP-OES as an increase in copper in mitochondrial pellets over time. Copper uptake was assayed in *L. lactis* using a modified method with whole cells resuspended in 2 µM coppersulfate or 2 µM iron-sulfate salts in water. Cells were incubated for different time points at room temperature, removed by centrifugation, washed in water, and total metals were measured by ICP-OES. Uptake was reported as the increase in copper or iron over time. The rates of copper or iron uptake were assessed in the linear portion of the uptake curves, normally over the first 5–10 min [19].

### 2.5. Expression of recombinant proteins

The *PIC2* and *MRS3* ORFs were cloned into pHis parallel 1. The fidelity of the construct was verified by dideoxynucleotide sequencing prior to use. BL21 (DE3) cells were transformed with the vector, and protein expression was induced with isopropyl *β*-d-1-thiogalactopyranoside for 3 h. Inclusion bodies were isolated in a manner similar to that described by Palmieri *et al.* [21]. Briefly, cells were resuspended in potassium phosphate buffer (140 mM NaCl, 2.7 mM KCl, 8.3 mM K_2_HPO_4_, 1.8 mM KH_2_PO_4_), pH 7.5, and disrupted by sonication. Insoluble material was collected by centrifugation at 18 500*g*. Insoluble material was resuspended in potassium phosphate buffer, pH 7.5, and loaded onto a stepwise 40%, 53%, 70% sucrose gradient. Samples were centrifuged at 18 500*g* for 1 h, and inclusion bodies were isolated as a grey-coloured band at the interface between the 53% and 70% layers. Presence of the protein of interest was confirmed by immunoblot. To load protein into liposomes, inclusion bodies were solubilized in 6 M urea before overnight dialysis in egg-yolk phospholipids dispersed in potassium phosphate buffer. The dialysed mixture was sonicated to generate the final proteoliposomes, and protein concentration was determined by Bradford assay.

### 2.6. Fluorescence anisotropy

CuL was diluted to give a fluorescence intensity of approximately 30 using an excitation at 320 nm and emissions at 400 nm. MCF protein incorporated into liposomes, or mitochondrial membranes isolated by sonication were added in 1–5 µl increments. Protein concentrations were determined by Bradford assay. Anisotropy was measured using a PerkinElmer Life Sciences LS55 spectrofluorimeter.

### 2.7. Miscellaneous methods

The monoclonal mouse anti-human SOD1 was purchased from Santa Cruz Biosciences. Secondary antibodies used were Cy3-linked goat anti mouse from GeneScript. Superoxide dismutase (SOD1) activity was measured using a xanthine oxidase linked assay kit (Sigma Life Science), and absorbance was measured on a BioTek 96-well plate reader.

## 3. Results

### 3.1. Simultaneous deletion of *PIC2* and *MRS3* results in copper-dependent growth defects

**Figure 1.**
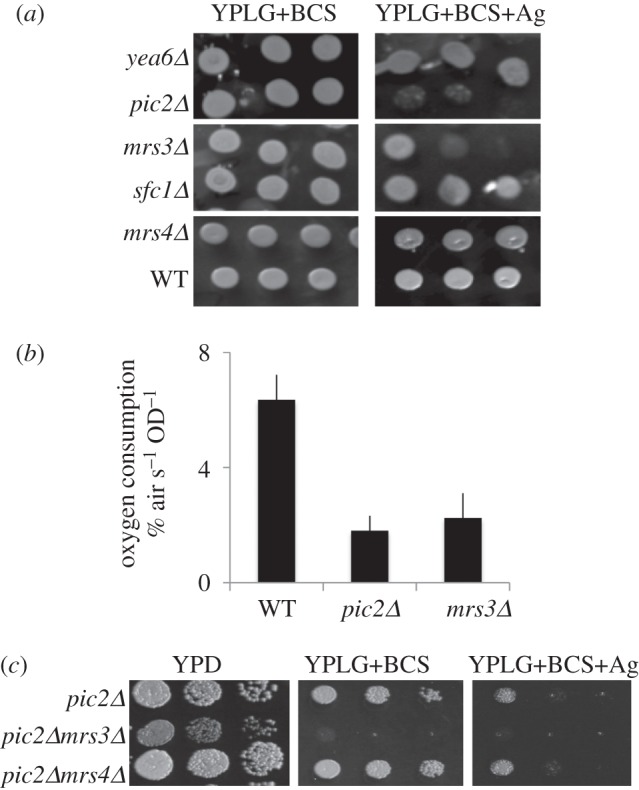
Copper-related growth phenotypes in *pic2Δmrs3Δ*. (*a*) Serial dilutions of BY4741 (wild-type; WT) and single MCF deletion strains grown on rich medium with a non-fermentable carbon source (lactate–glycerol) in the presence of the cell-impermeable copper chelator bathocuproine sulfonate (YPLG+BCS) or the same medium with supplemental silver at 100 µM (YPLG+BCS+Ag). (*b*) Oxygen consumption of whole WT, *pic2Δ* or *mrs3Δ* cells grown in rich medium with glucose and 50 µM silver added. (*c*) Serial dilutions of double mutant strains indicated grown on rich medium with a fermentable (glucose; YPD) or a non-fermentable carbon source (lactate–glycerol) in the presence of the cell-impermeable copper chelator BCS (YPLG+BCS) or in the presence of BCS and silver.

Pic2 was identified using a screen of single MCF gene deletions grown on rich medium with a non-fermentable carbon source in the presence of the cell-impermeable copper chelator BCS and silver as a mitochondrial copper competitor [19]. Under these conditions, the *mrs3Δ* mutant had a growth defect, though it was milder than a *pic2Δ* mutant (figure 1*a*). Yeast mutants with deletions in other MCF proteins, including the nicotinamide adenine dinucleotide transporter (*YEA*6), the succinate–fumarate exchanger (*SFC1*) or the *MRS3* paralogue *MRS4*, which encodes another component of the iron import machinery, did not show the same sensitivity to silver (figure 1*a*). The *mrs3Δ* growth defect was reversed when silver concentrations were decreased, and no defect was observed on glucose at the same concentration of silver (not shown). In addition, oxygen consumption was decreased in *pic2Δ* and *mrs3Δ* cells compared with wild-type when cultured in rich medium with glucose and 50 µM silver (figure 1*b*). To test for synergistic phenotypes, we generated a *pic2Δmrs3Δ* double mutant. Simultaneous deletion of *PIC2* and *MRS3* resulted in poor growth on copper-depleted rich medium with a non-fermentable carbon source (figure 1*c*) and on synthetic medium with a non-fermentable carbon source in the absence of chelators (not shown). Deletion of *MRS4* in the *pic2Δ* background did not cause an additional defect when compared with *pic2Δ* alone under any conditions tested (figure 1*c*).

**Figure 2.**
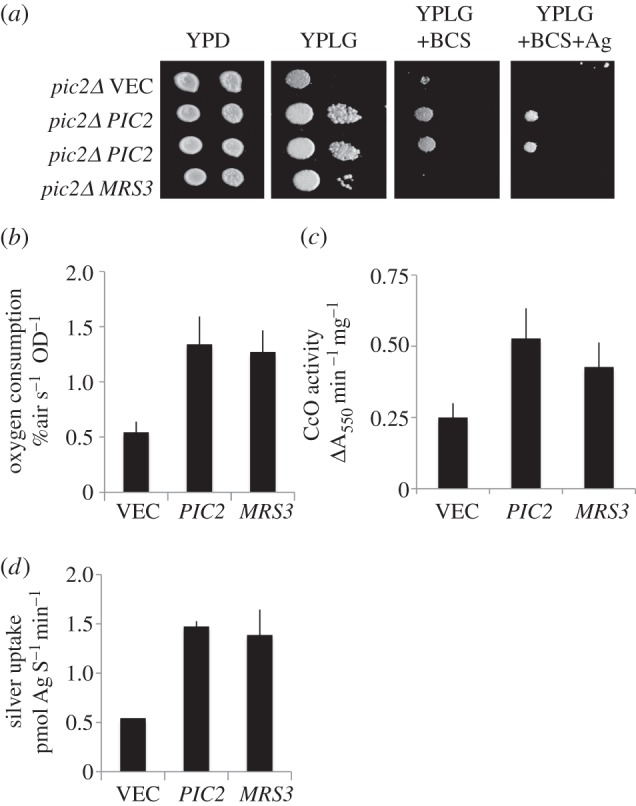
Suppression of *pic2Δ* by *MRS3*. (*a*) Serial dilutions of *pic2Δ* strains transformed with episomal vector containing no insert (VEC), or *PIC2* cloned under the control of the endogenous promoter (*PIC2*), *PIC2* under the control of high expression *ADH1* promoter or *MRS3* under the control of the endogenous promoter grown on rich medium with a fermentable carbon source (dextrose; YPD), non-fermentable carbon source (lactate–glycerol; YPLG), non-fermentable carbon source in the presence of the cell-impermeable copper chelator BCS (YPLG+BCS) or the same medium with supplemental silver at 100 µM (YPLG+BCS+Ag). (*b*) Oxygen consumption of whole *pic2Δ* cells with either empty vector, multi-copy vector with *PIC2* under control of the endogenous promoter or multi-copy vector with *MRS3* under control of the endogenous promoter. (*c*) Mitochondria isolated from strains in (*b*) assayed for cytochrome *c* oxidase (CcO) activity normalized to total protein. (*d*) AgL uptake into isolated mitochondria from (*c*) initial rates calculated from first 30–60 s of uptake at 10°C as described previously [19].

To test for the rescue of copper defects in the *pic2Δ* mutant by *MRS3,* we transformed the deletion strain with a high copy vector containing *MRS3*. Overexpression of *MRS3* weakly suppressed the growth defect of a *pic2Δ* mutant on non-fermentable carbon sources but only in the absence of silver (figure 2*a*). Consistent with the growth phenotype, *pic2Δ* cells with vectors containing either *PIC2* or multi-copy *MRS3* grown in the absence of silver had increased oxygen consumption (figure 2*b*). Mitochondria prepared from these cells had increased cytochrome *c* oxidase activity (figure 2*c*) and were able to support increased *in vitro* uptake of the copper analogue silver (provided as isolated AgL) when compared with *pic2Δ* mitochondria (figure 2*d*). These results suggest that *PIC2* and *MRS3* are both capable of copper import to support respiratory growth in yeast.

### 3.2. Deletion of *PIC2* and *MRS3* results in mitochondrial copper deficiency

**Figure 3.**
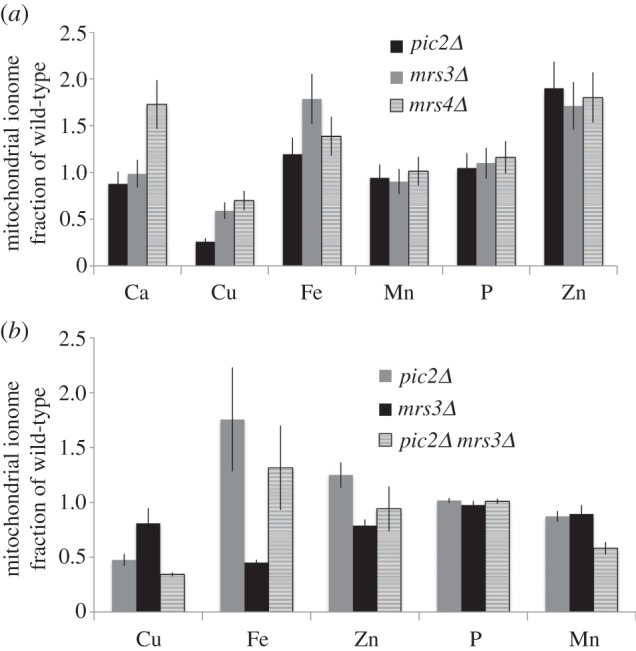
Total mitochondrial metals in *pic2Δ, mrs3Δ, mrs4Δ* and *pic2Δmrs3Δ*. (*a*) Relative concentrations of Ca, Cu, Fe, Mn, P and Zn in mitochondria from *pic2Δ, mrs3Δ* and *mrs4Δ* cells grown in rich medium with a fermentable carbon source (glucose) with 0.5 mM FeSO_4_. (*b*) Relative concentrations of Cu, Fe, Zn, P and Mn in mitochondria from *pic2Δ* (*n* = 24)*, mrs3Δ* (*n* = 14) and *pic2Δmrs3Δ* (*n* = 6) cells grown in synthetic medium with a fermentable carbon source (glucose) with added 0.5 mM CuSO_4_ and 0.5 mM FeSO_4_. Copper concentrations significantly decreased in *pic2Δmrs3Δ* compared with *pic2Δ* based on two-tailed *t*-test (*p* = 0.03). Metals were measured by ICP-OES and normalized on a per-sulfur basis before being calculated as a fraction of those found in wild-type cells.

To investigate the effects of loss of *PIC2* and *MRS3* on total mitochondrial copper, we isolated intact purified mitochondria for analysis by ICP-OES. Mitochondria isolated from *pic2Δ*, *mrs3Δ* and *mrs4Δ* cells grown in rich medium supplemented with iron showed copper levels decreased to 0.3, 0.6, 0.7 as a fraction of wild-type, respectively (to calculate fraction of wild-type, all values were divided by average wild-type concentration, so that equal levels would be 1 and a decrease is less than 1.0; figure 3*a*). To exaggerate changes in mitochondrial copper levels, wild-type, *pic2Δ* and *mrs3Δ*, and *pic2Δmrs3Δ* cells were grown in synthetic medium supplemented with 0.5 mM CuSO_4_. Both *pic2Δ* and *mrs3Δ* mitochondria accumulated copper to 0.45 and 0.8 as a fraction of wild-type mitochondria, respectively (figure 3*b*). The *mrs3Δ* mitochondria showed an iron defect, whereas *pic2Δ* mitochondria had a copper-specific defect. The *pic2Δmrs3Δ* double mutant accumulated only about 0.3 as a fraction of wild-type copper and 0.6 as a fraction of wild-type manganese, whereas other metals remained at or greater than wild-type levels (figure 3*b*). As previously observed [19], all mutants expanded the mitochondrial copper pool when grown with added exogenous copper, but expansion was attenuated relative to wild-type cells. Taken together, these results suggest that both Pic2 and Mrs3 have overlapping functions in maintaining mitochondrial copper pools and suggest the existence of an additional transporter that maintains mitochondrial copper in the double deletion.

### 3.3. *MRS3* can mediate copper uptake

**Figure 4.**
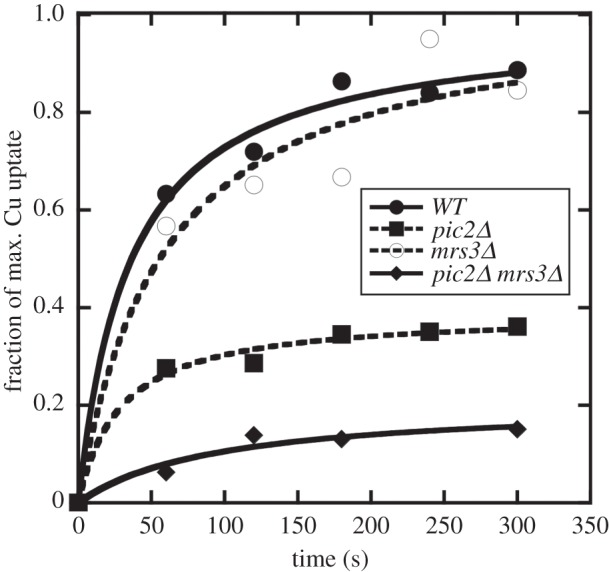
Mitochondrial copper uptake in WT, *pic2Δ, mrs3Δ* and *pic2Δmrs3Δ*. Isolated mitochondria from parental, *mrs3Δ, pic2Δ* or *pic2Δmrs3Δ* cells assayed for *in vitro* uptake of the CuL as measured by an increase in copper over time. Uptake is reported as the percentage of maximum uptake observed in wild-type mitochondria. Representative data are shown of three repeated measurements of uptake. Mitochondria were normalized for protein, and copper was measured by ICP-OES and normalized on a per-sulfur basis.

To determine the role for Mrs3 in mitochondrial copper maintenance, intact mitochondria from *pic2Δ*, *mrs3Δ* and wild-type cells were assayed for uptake of copper in the form of purified CuL. Copper uptake was decreased in *pic2Δ* mitochondria, whereas uptake into *mrs3Δ* mitochondria was not changed relative to wild-type (figure 4). However, simultaneous deletion of both *PIC2* and *MRS3* resulted in a further decrease of copper uptake relative to deletion of *PIC2* alone (figure 4). After uptake mitochondria were lysed by sonication and the soluble and insoluble fractions separated by centrifugation, more than 85% of the copper was released in the soluble fraction suggesting that the majority of copper accumulation was not due to binding to membranes (not shown) and performing assays at 4°C inhibited uptake [19]. These data indicate that simultaneous deletion of *PIC2* and *MRS3* causes a defect in mitochondrial copper import.

**Figure 5.**
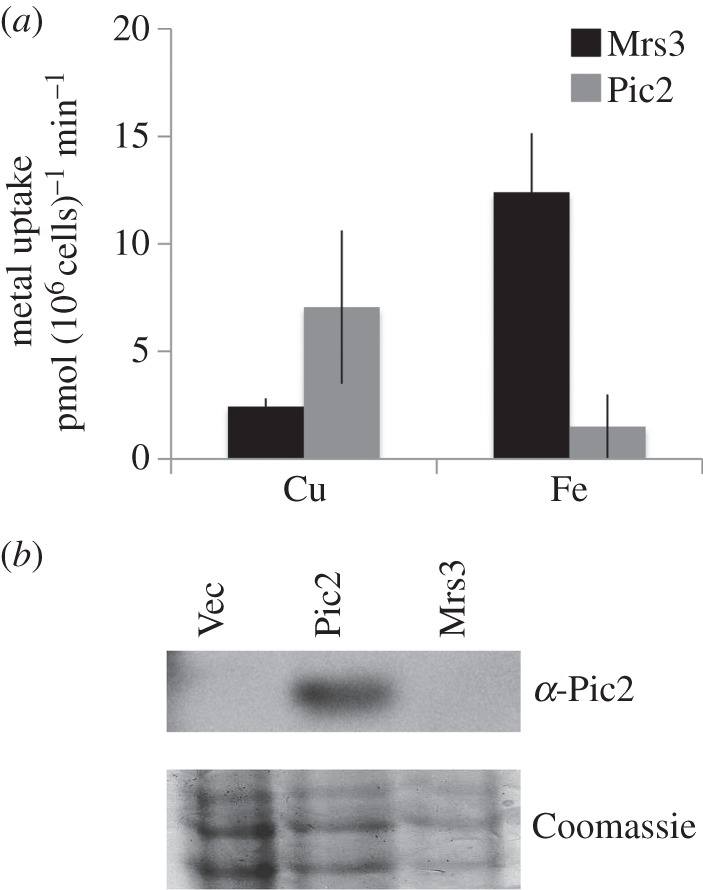
Expression of Mrs3 in *Lactococcus lactis*. (*a*) Uptake of copper or iron by intact cells transformed with *MRS3* or *PIC2* incubated at room temperature for 10 min with 2 µM CuSO_4_ (*n* = 4) or 2 µM FeSO_4_ (*n* = 4). Uptake observed in cells expressing empty vector was subtracted and rate increase per minute was calculated on a per 10^6^ cell basis. Error bars represent standard deviation. (*b*) Western blot of *L. lactis* transformed with empty vector (Vec) or *PIC2* or *MRS3* probed with antibody specific to Pic2 and an identical gel with the same samples loaded stained with Coomassie as a loading control.

To measure copper import in a heterologous system, *MRS3* was expressed in the Gram-positive bacterium *Lactococcus lactis*, which has been previously used to demonstrate function of MCF proteins, including copper uptake by Pic2 [19,33]. Uptake of CuSO_4_ was assayed in whole cells containing vector plus *MRS3* or cells containing empty vector. *L. lactis* cells expressing Mrs3 increased total copper, provided as CuSO_4_ (figure 5*a*). Both Pic2 and Mrs3 are expressed at low levels in *L. lactis* (less than 1% of total protein based on previous examples) [34] as they are not easily detectable by Coomassie staining after SDS–PAGE of total cell extracts [34]. Pic2 was detectable by immunoblot (figure 5*b*) [19], but no antibody was available to detect the level of expression of Mrs3. However, cells expressing Mrs3 were capable of importing FeSO_4_, which is consistent with the role of this protein in the iron import pathway and suggests that functional Mrs3 protein was expressed in *L. lactis* (figure 5*a*).

**Figure 6.**
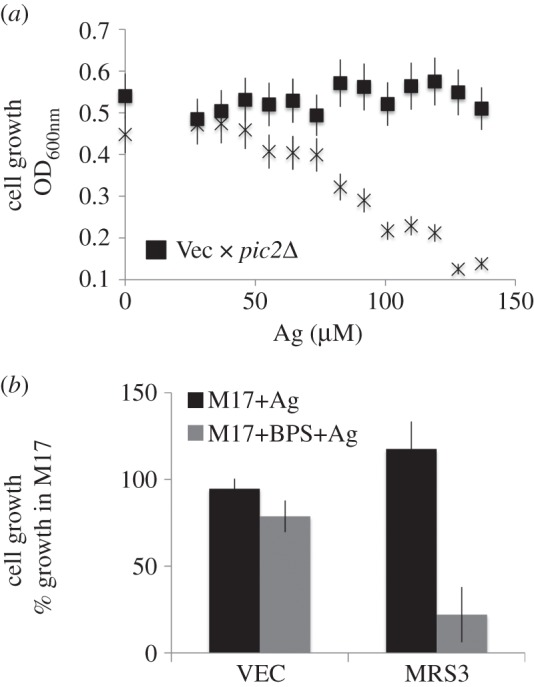
Silver toxicity in *Lactococcus lactis*. (*a*) Optical density at 600 nm of *L. lactis* carrying empty vector (filled square) and *PIC2* (cross) grown for 12 h in nisin to induce gene expression in the presence of increasing concentrations of silver. (*b*) Cell growth of *L. lactis* expressing *MRS3* or empty vector (VEC) grown in 120 µM silver with nisin in standard medium (M17) or in iron-depleted medium (M17+BPS).

Silver acts as a toxic copper mimetic that can be imported by copper carriers [19] and can be used to avoid obscuring of import results by endogenous copper. Thus, we designed a silver toxicity growth assay in *L. lactis* that was dependent on the expression of a functional expression of a copper importer. We observed dose-dependent silver toxicity in *L. lactis* when Pic2 expression was induced with nisin (figure 6*a*). No toxicity was observed in cells containing empty vector (figure 6*a*) or in *PIC2*-containing cells after being dosed with 120 µM silver in the absence of the inducer nisin (not shown), demonstrating that toxicity requires the expression of Pic2. Using this assay, we found that expression of Mrs3 did not induce silver toxicity even at 120 µM silver, indicating the lack of silver import (figure 6*b*). Given the iron transport function of Mrs3, we hypothesized that the iron concentration in the medium may be sufficient to compete with silver for import. Therefore, we assayed for silver toxicity under conditions where iron availability was decreased by addition of an extracellular chelator. Under iron-limiting conditions, silver was toxic to cells containing *MRS3* relative to those that contained an empty vector (figure 6*b*). These results indicate that Mrs3 is capable of transporting silver in *L. lactis* especially under iron-limiting conditions and suggest that Mrs3 can function as a copper importer.

### 3.4. Mrs3 can interact with the CuL complex

**Figure 7.**
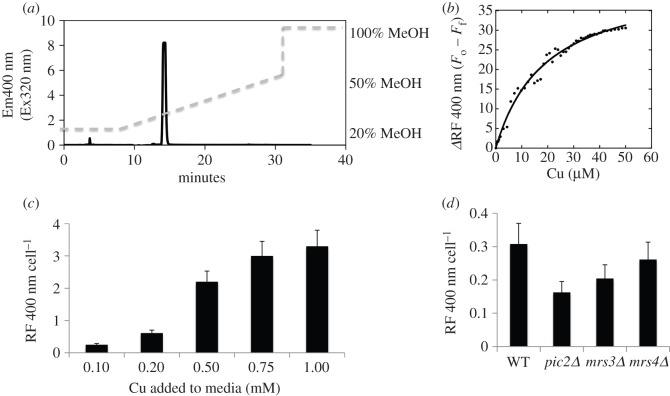
Fluorescence emissions of CuL with 320 nm excitation. (*a*) Reverse phase purification of anionic, copper-containing soluble matrix contents. Cells were grown overnight in SC (glucose) medium. Soluble contents were separated by anion exchange chromatography and loaded onto a C18 reverse phase column. CuL was observed as the fluorescent peak (Ex320 Em400) that eluted at 14 minutes (approx. 35% methanol). (*b*) Change in relative fluorescence (*Δ*RF) in CuL fractions titrated with sequential addition of Cu-acetonitrile. (*c*) CuL was prepared as in (*a*) from wild-type cells grown in SC medium with increasing CuSO_4_. The area of the 14-min ligand peak was quantified. Shown are the average intensities from three separate experiments. (*d*) Ligand was quantified from wild-type, *pic2Δ, mrs3Δ* and *mrs4Δ* cells grown in YPD media with 0.5 mM FeSO_4_. Shown is the average of three separate experiments. Error bars represent standard deviation.

The CuL complex in the mitochondrial matrix can be extracted in organic solvents, isolated with anion exchange resin, and fractionated and quantified by reverse phase chromatography. The final purified fraction has a fluorescent emission at 360 nm when excited at 220 nm that is responsive to the addition or removal of copper [20]. To analyse potential CuL–protein interactions, we used fluorescence anisotropy (FA) which measures the rotational diffusion of a molecule, so when the reactant binds its partner the larger product has a lower rotational diffusion coefficient and a higher FA. However, to avoid interference from tryptophan residues in added proteins, we scanned the excitation and emission profiles of the CuL to find a unique emission that could be excited with minimal interference. Purified CuL had a fluorescent emission at 400 nm upon excitation at 320 nm that could be detected as a single peak by reverse phase chromatography (figure 7*a*). As previously reported, fluorescence of the purified molecule was quenched in a concentration-dependent manner upon addition of Cu–acetonitrile (figure 7*b*). Addition of KCN to remove copper from the complex restored fluorescent emission. Upon copper supplementation to the medium, this peak increased (figure 7*c*), as did total mitochondrial copper (not shown). We compared the accumulation of the CuL complex in *pic2Δ*, *mrs3Δ* and *mrs4Δ* cells grown in rich medium with supplemental iron and detected a defect similar to that of mitochondrial copper in the same mutants (figures 3*a* and 7*d*).

**Figure 8.**
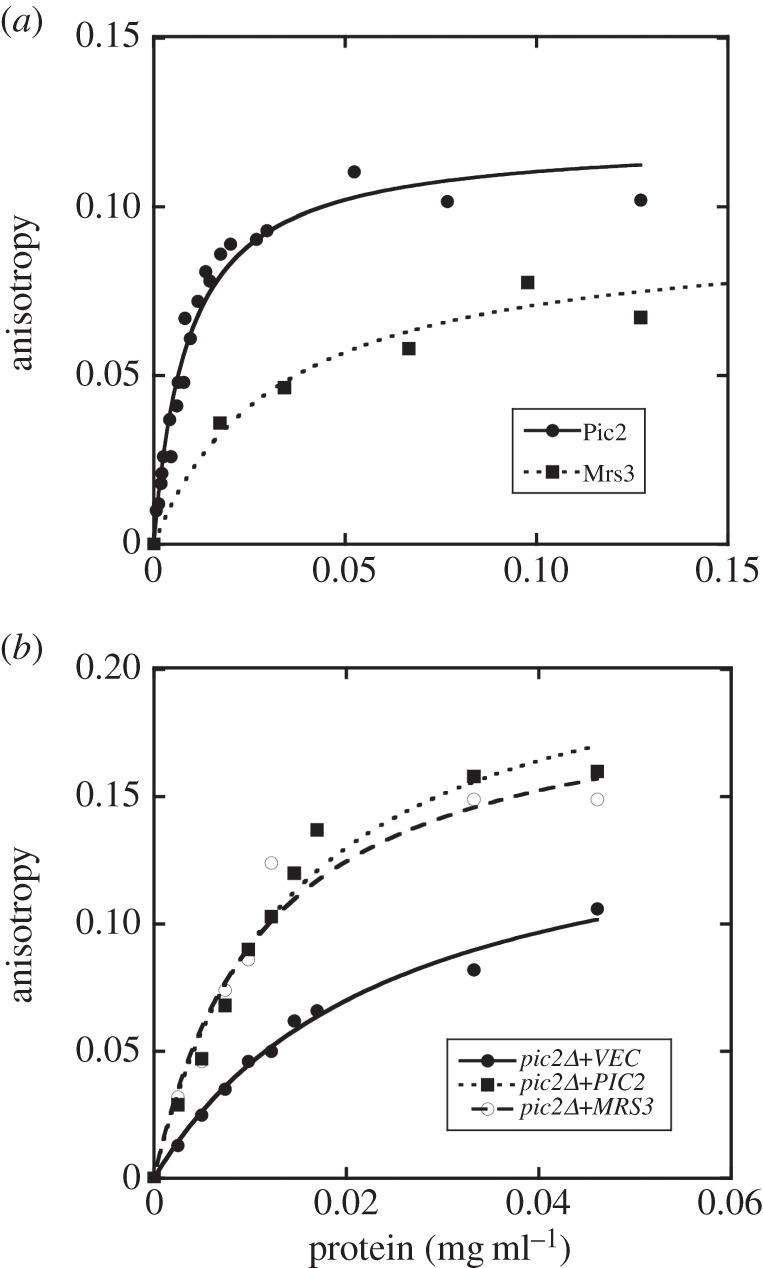
Anisotropy of CuL in presence of Pic2, Mrs3 and mitochondrial membranes. (*a*) Fluorescence anisotropy (FA) of CuL (Ex320 Em400) with addition of reconstituted Pic2 or Mrs3 in proteoliposomes prepared from extracted egg-yolk lipids. Control FA of equal quantity of lipids without protein added was subtracted from each point. (*b*) FA of CuL with mitochondrial membranes prepared by sonication of intact mitochondria followed by centrifugation from *pic2Δ* yeast expressing empty vector (VEC), *PIC2* or *MRS3.* Protein concentrations were determined by Bradford assay, and curves are fit with a hyperbola function.

We used FA to assay interaction between the Pic2 and the CuL complex. Pic2-containing liposomes were assayed for CuL binding by FA using the excitation/emission (320/400 nm) fluorescence characteristics of the CuL complex. Pic2 strongly enhanced anisotropy of the CuL, indicative of binding (figure 8*a*). Similarly, recombinant Mrs3 showed an interaction with CuL that was greater than liposomes alone (figure 8*a*). To support the data from recombinant proteins and avoid the requirement for refolding of Pic2 and Mrs3 in liposomes, we tested the binding of the CuL to membrane fractions from mitochondria. Membranes were prepared from intact mitochondria by sonication and centrifugation. The membrane fraction from *pic2Δ* mitochondria showed weak interaction with the CuL, whereas mitochondrial membranes isolated from *pic2Δ* expressing *PIC2* or *pic2Δ* with *MRS3* overexpression showed an enhanced interaction with the CuL as suggested by higher FA (figure 8*b*). These results suggest that both Pic2 and Mrs3 can interact with the CuL complex.

### 3.5. Heterologous copper protein activity

**Figure 9.**
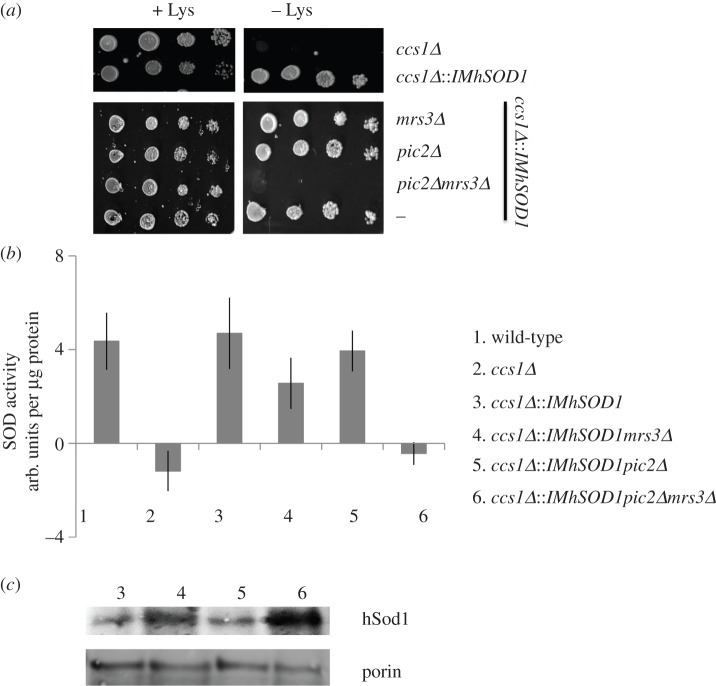
Deletion of *PIC2* and *MRS3* in a copper reporter strain. (*a*) Serial dilutions of wild-type and *ccs1Δ, ccs1Δ::IMhSOD1, mrs3Δccs1Δ::IMhSOD1, pic2Δccs1Δ::IMhSOD1* and *pic2Δmrs3Δccs1Δ::IMhSOD1* cells grown on synthetic medium under hyperoxic conditions with a fermentable carbon source (glucose) in the presence or absence of lysine. (*b*) Activity of SOD1 in isolated mitochondria from wild-type *(#1)*, *ccs1Δ* *(#2), ccs1Δ::IMhSOD1 (#3), mrs3Δccs1Δ::IMhSOD1 (#4), pic2Δccs1Δ::IMhSOD1 (#5),* and *pic2Δmrs3Δccs1Δ::IMhSOD1(#6)* cells grown in synthetic medium with fermentable carbon source as measured by xanthine oxidase/tetrazolium salt assay (*n* = 3) and normalized to total protein. (*d*) Immunoblot of hSOD1 and porin as a loading control from *ccs1Δ::IMhSOD1 (#3), mrs3Δccs1Δ::IMhSOD1(#4), pic2Δccs1Δ::IMhSOD1 (#5)* and *pic2Δmrs3Δccs1Δ::IMhSOD1 (#6)*.

To further demonstrate the role for Mrs3 in mitochondrial copper availability, we used a strain of *S. cerevisiae* that encodes an IM-tethered human Sod1, which acts as a mitochondrial copper sensor [20]. Use of this strain allows for growth-based assays of *mrs3Δ* using a phenotype that is not affected by its role in iron transport. In *S. cerevisiae*, deletion of the gene encoding the copper chaperone for Sod1 (*CCS1*) causes a loss of Sod1 activity resulting in a lysine auxotrophy [35,36]. The lysine auxotrophy of *ccs1Δ* can be reversed by expression of IM-tethered human SOD1 (IM-hSod1), and activity of this enzyme is dependent on available copper in the IMS (figure 9*a*) [20]. Because copper used by IMS cuproenzymes originates from the matrix pool, Sod1 activity and lysine prototrophy in the *ccs1Δ::IM-hSOD1* background can be used to probe availability of both matrix and IMS copper. Deletion of *PIC2* in this background caused a 40–50% decrease IM-hSod1 activity when these cells were grown in silver, but did not cause a lysine auxotrophy [19]. The *ccs1Δ::IM-hSOD1* copper-sensing strain was crossed with the *mrs3Δ* single mutant to generate the *mrs3Δccs1Δ::IM-hSOD1* and the *pic2Δmrs3Δ* double mutant to generate a *pic2Δmrs3Δccs1Δ::IM-hSOD1* triple mutant. While the double mutant *mrs3Δccs1Δ::IM-hSOD1* did not have any growth defects, the triple mutant *pic2Δmrs3Δccs1Δ::IM-hSOD1* failed to grow on medium lacking lysine (figure 9*a*). The triple mutant completely lacked Sod1 activity, indicating a deficiency of available mitochondrial copper, whereas the *mrs3Δccs1Δ::IM-hSOD1* double mutant had a mild deficiency of hSod1 activity (figure 9*b*). All strains expressed stable levels of IM-hSod1 (figure 9*c*). These results reveal that *MRS3* affects availability of copper in mitochondria.

## 4. Discussion

Copper, iron, manganese and zinc are all found in the mitochondrial matrix, yet understanding of the pathways by which they cross the IM remains incomplete. MCF proteins transport a large number of substrates, including metals, across the sealed mitochondrial IM. The identity of the substrates has been carefully and elegantly studied using multiple strategies, including phenotypic characterization, metabolomic analysis and import assays using MCF proteins heterologously expressed in *L. lactis* or purified and reconstituted into liposomes [37]. These approaches not only have identified many of the primary substrates for this family, but have also shown that a single MCF protein can have multiple substrates and that paralogous MCF proteins can have significantly different affinity for the same substrate. Thus, it is possible that even when an MCF has a defined substrate, it may be able to transport other substrates with equal or even better affinity or maximal rates. This apparent redundancy of some MCFs means that a single deletion may not result in a strong phenotype and additional measures or deletions are required to induce phenotypes.

We report that *MRS3*, which encodes a mitochondrial inner-membrane iron transporter, is also involved in mitochondrial copper homeostasis. *In vivo* phenotypes reported here support Mrs3 having partially overlapping function with the copper transporter Pic2. Simultaneous deletion of *PIC2* and *MRS3* exaggerates multiple copper-related phenotypes: (i) a more severe copper-dependent respiratory defect; (ii) defects in expansion of the mitochondrial copper pool; and (iii) failure to activate a copper-sensing IM-tethered hSod1. The exaggerated defects associated with *pic2Δmrs3Δ* suggest that these two proteins function independently in the copper import pathway. These observations are supported by the fact others have reported copper uptake defects in mitochondrial inner-membrane vesicles lacking Mrs3 and Mrs4 (18).

We suggest a model where Mrs3 acts as a required copper importer *in vivo* under certain copper-limiting conditions or in the absence of Pic2. Deletion of *MRS3* alone does not appear to dramatically affect the total mitochondrial copper content or copper uptake in intact mitochondria, yet Mrs3 expressed in *L. lactis* can mediate copper import. This result may indicate that the steady-state assays of total copper in mitochondria cannot capture a subtle change associated with the loss of *MRS3*. Additionally, the purified mitochondria used in these analyses are selected by the centrifugation and density gradients and may not include the total pool of mitochondria. Further studies are needed to define the exact function of Mrs3 in the mitochondrial copper import pathway in yeast. Both Pic2 and Mrs3 have orthologues in multicellular eukaryotes (e.g. in humans *SLC25A3* for *PIC2,* and *SLC25A37* and *SLC25A28* for *MRS3*), and mutation of these genes causes diseases associated with loss of transport of phosphate and iron [25,30,38,39]. It remains to be investigated whether these homologues have copper transport activity and how this transport is involved in normal physiology.

This report is the first evidence of an interaction between the CuL and Pic2 and Mrs3, which is consistent with the model that the CuL is the substrate for both carriers. Transport of this ligated form of copper probably helps prevent copper from negatively interacting with other mitochondrial contents. For example, Foster *et al.* [40] demonstrated that copper, supplied in the presence of 2-(6-benzyl-2-pyridyl) quinazoline (BPQ), accumulated at high levels in the cell, including mitochondria, and disrupted Fe–S clusters. Disruption of Fe–S synthesis by copper accumulation may present a pressure to avoid excess or free copper in mitochondria. Thus, if Cu were to accumulate independently of the CuL complex, then this protection would be lost. Interestingly, we found that Pic2 and Mrs3 were capable of transporting ionic copper and silver as seen in the silver toxicity assay in *L. lactis.* Therefore, Pic2 or Mrs3 may be associated with potential copper toxicity in mitochondria. Sequence alignment of Pic2 and Mrs3 and the closely related Mrs4 combined with structural modelling did not suggest obvious conserved residues that would impart copper transport by Mrs3 but not Mrs4. We plan to use the silver toxicity assay to define the residues required for transport of copper versus CuL and the differences between Mrs3 and Mrs4.

In conclusion, we report phenotypic and biochemical evidence for the involvement of Mrs3 in mitochondrial copper import. The results increase our understanding of mitochondrial copper import and will lead to additional studies to better understand the mechanisms of copper import, the identity of copper-exporting carriers and connections between copper and iron homeostasis in mitochondria.
